# The Soluble Form of the EIAV Receptor Encoded by an Alternative Splicing Variant Inhibits EIAV Infection of Target Cells

**DOI:** 10.1371/journal.pone.0079299

**Published:** 2013-11-22

**Authors:** Yue-Zhi Lin, Fei Yang, Shu-Qin Zhang, Liu-Ke Sun, Xue-Feng Wang, Cheng Du, Jian-Hua Zhou

**Affiliations:** 1 Key Laboratory of Veterinary Biotechnology, Harbin Veterinary Research Institute, Chinese Academy of Agricultural Science, Harbin, China; 2 Institute of Special Wild Economic Animal and Plant Science, Chinese Academy of Agricultural Science, Changchun, China; National Institute of Allergy and Infectious Diseases, United States of America

## Abstract

Equine lentivirus receptor 1 (ELR1) has been identified as the sole receptor for equine infectious anemia virus (EIAV) and is a member of the tumor necrosis factor receptor (TNFR) superfamily. In addition to the previously described membrane-associated form of ELR1, two other major alternative splicing variant mRNAs were identified in equine monocyte-derived macrophages (eMDMs). One major spliced species (ELR1-IN) contained an insertion of 153 nt, which resulted in a premature stop codon situated 561 nt upstream of the predicted membrane spanning domain. The other major species (ELR1-DE) has a deletion of 109 nt that causes a shift of the open reading frame and generates a stop codon 312 nt downstream. Because ELR1-DE presumably encodes a peptide of a mere 23 residues, only ELR1-IN was further analyzed. The expression of a soluble form of ELR1 (sELR1) by ELR1-IN was confirmed by Western blot and immunofluorescence analyses. Similar to ELR1, the transcription level of ELR1-IN varied among individual horses and at different time points in the same individuals. The ratio of ELR1-IN mRNA species to ELR1 mRNA was approximately 1∶2.5. Pre-incubation of the recombinant sELR1 with EIAV significantly inhibited EIAV infection in equine macrophages, the primary in vivo target cell of the virus. Fetal equine dermal (FED) cells are susceptible to EIAV in vitro, and the replication of EIAV in FED cells transiently transfected with ELR1-IN was markedly reduced when compared with replication in cells transfected with the empty vector. Finally, the expression levels of both forms of the EIAV receptor were significantly regulated by infection with this virus. Taken together, our data indicate that sELR1 acts as a secreted cellular factor that inhibits EIAV infection in host cells.

## Introduction

For most retroviruses, the viral envelope binds to receptors in a pH-independent manner, suggesting that the virions can fuse directly to the cell membrane [1]. Therefore, viral receptors on the cell membrane provide binding sites for the virus and are also involved in the structural modulation of viral envelopes, leading to the fusion of the cellular and viral membranes and virion entry, the first step in viral infection of target cells [2]. Accordingly, studies of the role of viral receptors in the invasion of the virus are important to the development of antiviral reagents and vaccines.

The equine infectious anemia virus (EIAV) is a member of the genus Lentivirus, family Retroviridae, and its structure is the simplest out of all the known lentiviruses [3]. The receptor of EIAV is equine lentivirus receptor 1 (ELR1), which was identified by Zhang et al. in 2005 using a functional cloning approach [4]. In contrast to most other lentiviruses, such as human immunodeficiency virus (HIV)-1, simian immunodeficiency virus (SIV) and feline immunodeficiency virus (FIV), which require co-receptors for successful infection, EIAV appears to depend only on a functional ELR1 for the invasion of target cells. Based on its sequence and structural characteristics, ELR1 belongs to the TNF receptor (TNFR) superfamily [4], [5], and many receptors of this superfamily, such as the growth factor receptor, leptin receptor and Fas, also have soluble forms. Soluble forms have also been identified for some immunoglobulins and chemokine receptors [6]–[8].

Soluble receptors can be processed posttranscriptionally or posttranslationally. The release of membrane-associated forms from the cell surface contributes significantly to the formation of soluble receptors at the posttranslational level; this process is usually catalyzed by enzymes and highly is regulated. In addition, the alterative splicing of mRNAs during the maturation of eukaryotic pro-mRNA is another mechanism for the formation of soluble receptors. The translation of receptor mRNA can be prematurely terminated due to alterative splicing, which produces receptors that lack the transmembrane and cytoplasmic domains [6], [7], [9].

Much evidence has demonstrated that soluble viral receptors are functionally important for viral infections [10]–[13]. The soluble receptors for HIV-1, EBV (Epstein-Barr virus) and rhinovirus are reportedly able to inhibit infection by the corresponding viruses [11], [14], [15]. Another study found that the soluble form of the avian sarcoma leukosis virus subgroup A (ASLV-A) receptor Tva (sTva) inhibited the infectivity of this virus by 90% at a low concentration (25 pM) but mediated ASLV-A infection in cells lacking the receptor at a high concentration (5 nM) [16]. Brindley et al. demonstrated that preincubation of EIAV with the soluble ectodomain of ELR1 dramatically reduced the viral infectivity on the target cells [17]. These data further implicate soluble viral receptors in the interaction between viruses and their host cells.

As mentioned above, the investigation of the alternative splicing isoforms of a given receptor facilitates a better understanding of its functions. To the best of our knowledge, there are no reports on naturally expressed soluble EIAV receptors. Therefore, the present study had three objectives: 1) to identify alternative splicing variants for ELR1, 2) to determine whether any of these variants encode soluble ELR1 and 3) to characterize the roles of any soluble forms of the receptor in EIAV infection.

## Materials and Methods

### Ethics statement

Horses and related experimental protocols used in this study were approved by the Institutional Animal Care and Use Committee (IACUC) of the Harbin Veterinary Research Institute (HVRI), Chinese Academy of Agricultural Sciences. There were no animals scarified specifically for this study. Fetal equine dermal (FED) cells and 293T cells were proliferated from the stock of the Cell Bank of Epidemic Disease Examination and Service Center of HVRI. Equine monocyte-derived macrophages (MDMs) were prepared from peripheral blood mononuclear cells (PBMC) that were isolated from 200–300 ml horse peripheral blood taken from the jugular vein by veterinarians.

Horses were infected with EIAV in a previous study [19], and the animals infected with pathogenic EIAV strains were euthanized at the end of experiment or when demonstrated severe disease-associated symptoms resulting in distress by intravenous injection of Pelltobarbitalum Natricum (100 mg/kg of body weight, dissolved in saline) in the jugular vein by veterinarians according to protocols approved by IACUC of HVRI.

### Cells and strains

Fetal equine dermal (FED) cells, equine monocyte-derived macrophages (MDMs) and human 293T cells were used for the transfections and the expression of recombinant proteins. The cells were maintained in high-glucose Dulbecco's modified Eagle's medium (DMEM, Gibco, USA) supplemented with penicillin and streptomycin. The medium was supplemented with 10% fetal calf serum (FCS) for the FED and 293T cells. Equine MDMs (eMDMs) were enriched from whole blood as described previously [18]. EIAV_DLV34_ (DLV34) is a cell culture-adapted EIAV pathogenic strain, which was derived by 33 passages of the virulent EIAV_LN40_ strain in donkey MDMs. Similar to EIAV_LN40_, inoculation with 10^4^ TCID50 of DLV34 resulted in acute EIA in all the experimentally infected horses [19].

### Measurement of ELR1-IN mRNA expression by branched (b)DNA assay

The bDNA is a sandwich nucleic acid hybridization assay, in which target mRNA molecules are captured through cooperative hybridization of multiple probes. Unlike PCR, in which a region of the intended target is exponentially amplified in order to generate detectable signal, in bDNA assays only signals are amplified. bDNA assays are consequently not susceptible to contamination risks associated with PCR-based assays [20], [21]. Equine MDMs were cultured and ELR1-IN mRNA was extracted and quantified using bDNA assay as described previously [18]. Samples were read by a Luminex 200 (Molecular Devices, USA) and all data were analyzed using the Luminex IS2.3 program. An acquisition gate between 5000 and 20,000 was set to exclude any doublet events and ensure that only single microspheres were measured.

### The construction of vectors for the expression of HA- and GFP-tagged ELR1 and ELR1-IN

The ELR1 cDNA was cloned by reverse transcription PCR (RT-PCR) using equine MDM with primers based on the published sequence of equine ELR1 [4]. The green fluorescent protein (GFP) gene was subcloned from the pGFP-N3 vector (Clontech, USA), and the GFP-ELR1 fusion gene fragment was amplified by overlapping PCR. A human influenza hemagglutinin (HA) fragment was linked to the 3′ of the ELR1 cDNA for the detection of the expressed recombinant ELR1. The ELR1-HA fusion fragment was amplified using the primers 5′-GCGAATTCTTAGCACAGGGACGCGTAGTCCGGGACGTCGTATGGGTAGGCCTGGCAGCTCT-3′ for HA and 5′-GCGAATTCTTAGCACAGGGACGCGT AGTCCGGGACGTCGTATGGGTAGGTCTGAAGGAC-3′ for ELR1. Both of these PCR products were digested with *Eco*RI and *Hin*dIII and inserted into the pcDNA3.1 (+) vector (Invitrogen, USA) at the same cloning sites. Vectors for the expression of GFP- and HA-tagged ELR1-IN were constructed using the same procedures. Plasmids containing only the GFP or HA gene fragment were used as the controls for GFP and HA expression, respectively.

### Confirmation of soluble ELR1 (sELR1) expression

The translation of sELR1, i.e., the protein predicted to be encoded by ELR1-IN, was confirmed using Western blot and confocal imaging. Briefly, 293T cells were transfected with plasmids expressing HA-tagged ELR1 and ELR1-IN using PolyJet transfection reagents (Roche, USA). The expression of membrane-associated or soluble ELR1 was examined by Western blot using a monoclonal antibody against the HA tag (Sigma, USA) fused to the receptor at the C-terminus. Protein bands reacting with the HA antibody were visualized using a horseradish peroxidase (HRP)-conjugated goat anti-mouse antibody (Sigma, USA). To perform confocal imaging, 293T cells seeded on coverslips were transfected with the HA-tagged sELR1 or ELR1 expression vectors. After 72 h of incubation, transfected cells were fixed with 4% paraformaldehyde in PBS for 30 min and permeabilized with 0.1% Triton X-100 for 15 min. Cells were blocked with PBS/FISH GELATIN (5%) for 2 h, reacted with anti-HA monoclonal Ab for 1 h and then incubated with an anti-mouse IgG (whole molecule)-fluorescein isothiocyanate (FITC)-labeled antibody (F2012, Sigma). Cells were washed with PBS for three times and then stained with 4,6-diamidino-2-phenylindole (DAPI, Sigma) for 30 min and examined using a Leica TCS SP5 confocal system (Leica Microsystems, Germany). Appropriate IgG isotype controls were included to rule out non-specific binding. In addition, a 3D live cell imaging system (UltraView Vox, PerkinElmer, USA) was applied to observe the dynamically distribution of the sELR1 and ELR1 that fused to GFP at the N-terminus after being transiently expressed in 293T cells.

### In vitro EIAV infection inhibition assay

An in vitro EIAV infection inhibition assay was applied to examine the ability of sELR1 to interact with EIAV. Specifically, sELR1 was transiently expressed in 293T cells by transfection of the expression plasmid pcDNA3.1-ELR1-IN. The culture medium that contained the secreted sELR1 was collected at 48 h after transfection and incubated with 10^3^ TCID50 of the DLV34 strain at 4°C for 30 min. The viruses that were pre-incubated with sELR1 were then added to eMDMs in 6-well plates. Cell-free culture medium was collected at 1, 3, 5 and 7 days post-infection (dpi) and measured for viral reverse transcriptase (RT) activity using a Reverse Transcriptase Assay Colorimetric kit (Roche, Switzerland). The DLV34 strain incubated with the culture medium of cells transfected with the empty pcDNA3.1 vector was used as the control for non-specific bindings.

### The effect of sELR1 overexpression on the EIAV infection of target cells

sELR1 was transiently overexpressed in FED cells by transfection with pcDNA3.1-ELR1-IN using PolyJet transfection reagents. The FED cells were infected with 100 µl of appropriately diluted DLV34, 24 h after the transfection. The virion release into the medium was quantified at 2, 4, 6 and 8 dpi by measuring the viral RT activity.

### Quantitative analysis of ELR1 and ELR1-IN mRNA expression in cells infected with EIAV

The mRNA levels of ELR1 and ELR1-IN in equine MDMs infected with EIAV were determined using quantitative real-time PCR. Cultivated eMDMs were infected with 0.1 plaque-forming units (PFU) of DLV34 and collected at 4, 8 and 12 hours and 3 and 5 dpi. mRNA samples were prepared from these cells and reverse transcribed into cDNA using M-MLV Reverse Transcriptase (Invitrogen, USA), and the expression levels of ELR and ELR1-IN were then quantified using real-time PCR with a SYBR Green amplification and detection kit (QIAGEN, Germany). The expression levels of the ELR1 transcripts were normalized to that of the β-actin mRNA.

Plasmids containing either the ELR1 or ELR1-IN cDNA were constructed to establish standard curves for copy number determination. The ELR1 and ELR1-IN fragments were amplified by PCR, cloned into a pMD18-T vector and verified by DNA sequencing. The standard plasmid was serially diluted from 1×10^8^ to 1×10^2^ copies/ml and then measured using real-time PCR. For each reaction, 5 µl of standard plasmid or cDNA sample was added to a 20 µl reaction mixture containing 12.5 µl SYBR Green PCR Master mix and 3 µM of each primer. The amplification protocol was as follows: 95°C for 10 min, followed by 35 cycles at 95°C for 30 s, 60°C for 30 s and 65°C for 30 s; the final extension was at 68°C for 10 min. All the samples were measured twice. The ELR1 and ELR1-IN mRNA levels were calculated by dividing the receptor copy number by the β-actin copy number.

### Statistical analysis

Statistical analysis and data presentation were performed using Student's t-test (two-tailed, confidence intervals of 95%). and Graphpad Prism version 4.0 (GraphPad Software, USA) programs, respectively. A probability value less than 0.05 was considered statistically significant.

## Results

### The identification of alternative splicing variants for ELR1

**Figure 1:**
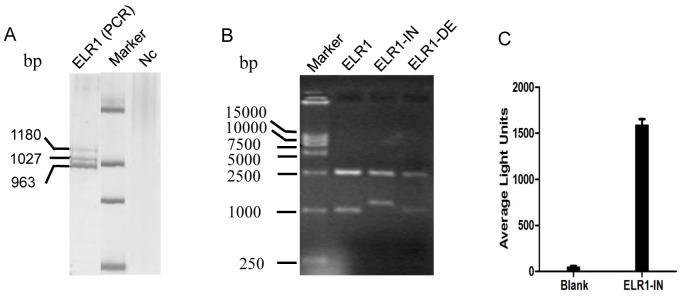
The identification of ELR1 splicing variants. ELR1 cDNA was amplified from mRNA templates extracted from eMDMs using reverse transcription PCR and then subcloned. (**A**) The amplified ELR1 cDNA fragments were separated by polyacrylamide gel electrophoresis and visualized by silver staining. (**B**) The subcloned inserts were examined by digesting the recombinant plasmids with the restriction enzymes *Eco*RI and *Hin*dIII, followed by agarose gel electrophoresis. Nc: the negative control sample for the PCR. (**C**) To confirm the presence of ELR1-IN, this species of mRNA in total RNA extracted from eMDM was further detected by hybridizing with a specific and branched probe using the bDNA technique. Data are the means of two independent experiments.

The ELR1 cDNA was amplified from mRNA templates extracted from eMDMs by reverse-transcription PCR using primers designed based on the sequence published in GenBank. Polyacrylamide gel electrophoresis revealed three bands migrating at approximately 1000 bp (Fig. 1A), and subcloning of the amplified fragments further confirmed the existence of three ELR1 cDNA fragments with different molecular masses (Fig. 1B). Nucleotide sequencing revealed two other species of ELR1: one containing an insertion of a 153 nucleotide (nt) fragment of intron 6 (ELR1-IN) and another with a deletion of 109 nt (ELR1-DE). To avoid the contamination of introns of chromosomal DNA, the ELR1-IN mRNA samples were pre-treated with DNase to remove any contaminating chromosomal DNA and then PCR amplified with a pair of primers, one of which targeted the insert. The 125 bp ELR1 cDNA fragment was amplified from both mRNA samples with or without DNase pre-treatment, indicating that ELR1-IN originated from the mRNA. As a control, we confirmed that a 501 bp fragment from intron 3 of the equine MHC-I gene was amplified from the DNA preparation without DNase treatment but not a preparation that was pre-incubated with the nuclease (Data not shown).

In addition, to exclude the possible artificial effect resulting in the ELR1-IN molecule during reverse transcription and PCR amplification, the presence of ELR1-IN mRNA was further measured by a bDNA assay, which detects specific mRNA species directly from total RNA preparations by hybridizing with signal-amplified probes specific for the inserted sequence without being revers transcribed and amplified. As shown in Fig. 1C, the average light unit of ELR1-IN mRNA was 1,594 while that of the blank control was 53.5.

### Analysis of ELR1 splicing variants

**Figure 2:**
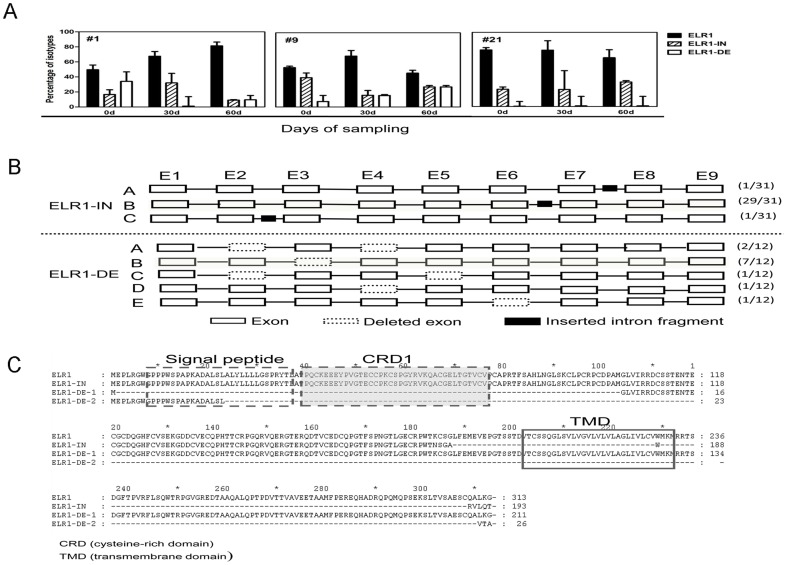
Dynamic changes of ELR1 splicing variants in hosts. ELR1 transcripts were amplified and subcloned from mRNA extracted from the PBMCs of three horses (#1, #9 and #21) at days 0, 30 and 60. A total of 126 subclones were randomly selected and sequenced. (**A**) The ratios of three major ELR1 splicing variants, ELR1, ELR1-IN and ELR1-DE, were analyzed and compared. (**B**) A schematic of the positions and relative frequencies of the three sliced forms of ELR1-IN and five ELR1-DE variants in 43 subclones sampled from three horses. The shaded form indicates the predominant isoform of ELR1-IN or ELR1-DE. E1 to E9 indicate exon 1 to exon 9, respectively. (**C**) The amino acid sequences of ELR1 and its splicing variants ELR1-IN and ELR1-DE were predicted and aligned. There are two presumed ORFs, ELR1-DE1 and ELR1-DE2, for the ELR1-DE isoform.

To further investigate the status of the ELR1 transcript variants in vivo, the variants were examined by cloning and sequencing the PCR-amplified specific cDNA fragments from the PBMCs of three horses collected at different time points. All three major variants of ELR1 mRNA were found in all the horses and at all the time points examined. However, the ratios of each isoform were varied among the different horses and at the different sampling times. Among the total 126 clones analyzed, the ELR1 species was predominant (83/126), followed by ELR1-IN (31/126) and ELR1-DE (12/126) (Fig. 2A). Furthermore, additional isoforms with insertions or deletions were identified. Among the 31 clones of ELR1-IN, 29 contained a fragment of intron 6, and the other two contained a fragment of intron 2 or intron 7. In addition, five types of deletions at different sites of the coding region were detected (Fig. 2B).

All the insertions and deletions resulted in a shift of the open reading frame (ORF). For the major form of ELR1-IN, the inclusion of a 153 nt fragment of intron 6 created an isoform with five different amino acid residues and then a premature stop codon 18 residues upstream of the predicted transmembrane domain (Fig. 2C). The cDNA of ELR1-DE, which lacked exon 3, contained two ORFs; the first encoded a peptide of only 27 residues, and the second encoded a receptor lacking the EIAV-binding CRD1 (Fig. 2C). Because both of these predicted proteins were considered not to function as EIAV receptors, they were not further evaluated in the present study.

### Conformation of a soluble EIR1 encoded by ELR1-IN

**Figure 3:**
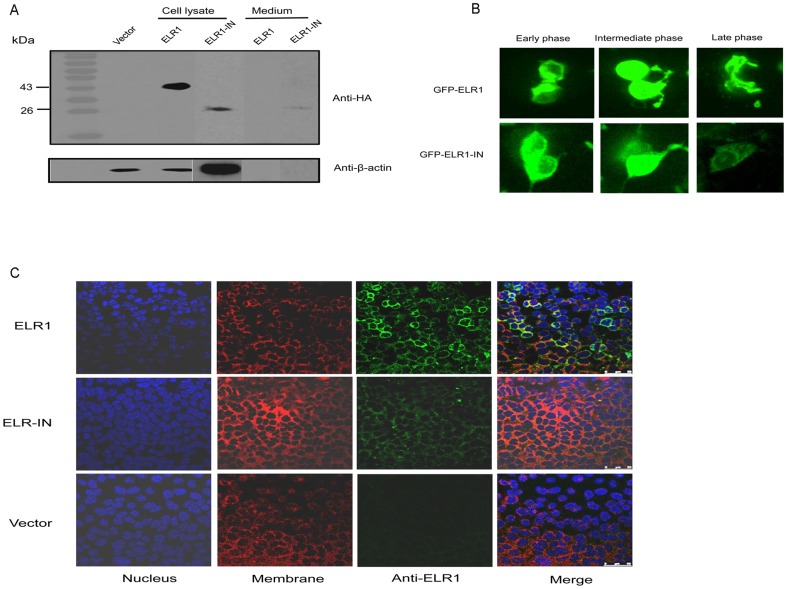
The translation of a soluble form of ELR1 from ELR1-IN. Three approaches were used to confirm the expression of the predicted sELR1. (**A**) The recombinant sELR1 was expressed as a fusion protein with an HA tag at the C-terminus in 293T cells, and its presence in the cell lysate or in the culture medium was examined by Western blot using an anti-HA antibody. β-actin was applied as the internal control protein. (B) Live cell imaging analysis of ELR1 and sELR1 expression in HEK 293T cells. These two forms of receptor were expressed as proteins with a GFP fused at the N-terminus of each. The three images of sELR1 were enhanced at a higher level than ELR1. (C) Cellular location of ELR1 and sELR-1. HA-tagged ELR1 and sELR1 were expressed in 293T cells for 72 h. Cells were then examined by confocal microscopy after being fixed, stained with DAPI for the nucleus (blue) and DII for the cell membrane (red) and reacted with an anti-HA antibody and a FITC-labeled anti-mouse IgG (green). Appropriate IgG isotype controls were included to rule out non-specific binding.

The receptor encoded by ELR1-IN was predicted to be a truncated form lacking the transmembrane domain and the cytoplasmic domain and, therefore, was presumed to be a soluble form of the receptor. The expression of soluble ELR1 (sELR1) was examined by Western blot, flow cytometry and confocal microscopy. An sELR1-HA fusion protein was overexpressed in 293T cells, and the 26 kDa sELR1-HA protein was detected in both the cell lysate and the culture medium by Western blot using an anti-HA antibody. As a control, membrane-associated ELR1-HA was detected only in the cell lysate (Fig. 3A).

In addition, both sELR1 and ELR1 were transiently overexpressed in 293T cells with green fluorescence protein (GFP) fused at the N-terminus. Flow cytometry analysis showed that, although the transfection efficiency was similar for both of the transfected cell populations, the fluorescence intensity of the GFP-sELR1- -expressing cells was markedly weaker than that of the GFP-ELR1-expressing cells, indicating a dramatic reduction in the intracellular accumulation of GFP-sELR1 relative to GFP-ELR1 (Fig. S1). The aforementioned experiment suggested that the EIAV receptor encoded by ELR1-IN was expressed as a soluble form of the receptor.

To further examine the dynamic cellular distribution of sELR1 and compare with that of ELR1, a live cell imaging of the dynamic expression and distribution of the GFP-ELR1 and GFP-sELR1 was taken. As shown in Fig. 3B, the membrane-associated GFP-ELR1 was accumulated as a few large spots in the cell plasma at the early phase post transfection (Fig. 3B). The fluorescence extended and covered to the whole cell surface and plasma thereafter (intermediate phase). At the late phase of cell life, the intracellular fluorescence was seen as bands and islands, which was considered the remaining membrane-bound ELR1 after the damage of plasma membrane. On the other hand, the transmembrane domain-free GFP-sELR1 was observed as diffused fluorescence, distributed like donuts at the early phase, evenly covered to the whole cell at the intermediate phase and gradually attenuated to weak and faint images at the late phase. At certain time points, the leak of fluorescent content from some cells could even be observed (see the video of 3D live cell image in Movie S1, e.g. at 05:00:00). These images vividly demonstrated the behaviors of GFP-sELR1 as a soluble protein.

The cellular location of ELR1 and sELR1 was also detected by confocal microscopy. These two forms of EIAV receptor were expressed as HA-tagged proteins in 293T cells and were detected by an HA monoclonal antibody and visualized by a FITC-labeled anti-mouse antibody. As displayed in Fig. 3C, the ELR1-HA was mostly detected on the cell membrane, so as the sELR1-HA, but at a much lower level. Combined with all the results submitted in Fig. 3 and Supplementary Materials, it indicates that sELR1 is expressed as a soluble protein and can be secreted out of the cell. However, some molecules of this soluble form of receptor are associated with the cell surface membrane.

### The effect of pre-incubation of sELR1 on EIAV infection

**Figure 4:**
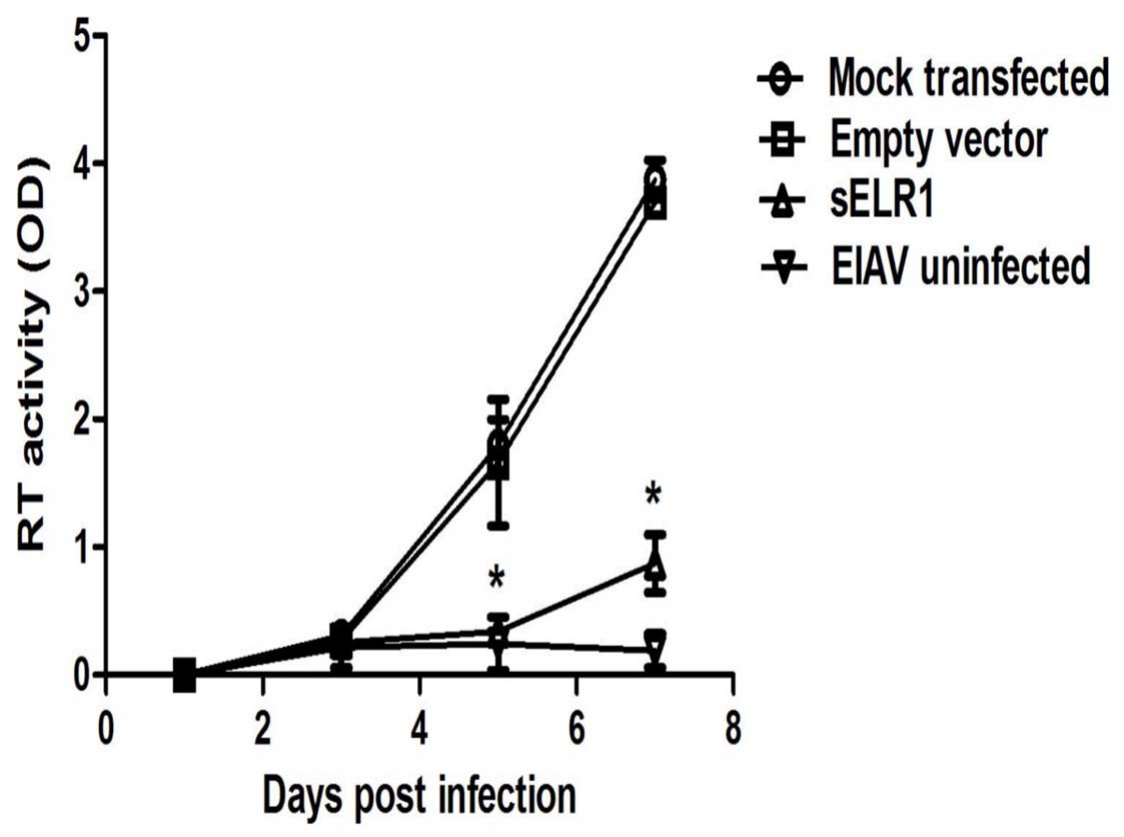
The effect of pre-incubation of sELR1 with EIAV on the viral infectivity. To examine the ability of sELR1 to competitively inhibit the infection of EIAV to target cells, the soluble receptor was overexpressed in 293T cells by transfection with the pcDNA3.1-ELR1-IN expression vector for 48 hours. The culture medium, containing secreted sELR1, was incubated with an EIAV strain DLV34 at 4°C for 30 min. Virus that had been pre-incubated with sELR1 was used to infect eMDMs in 6-well plates. DLV34 pre-incubated with medium from cells that had been transfected with the empty vector pcDNA3.1 or untransfected cells were also used to infect eMDMs as controls. EIAV replication was monitored by measuring viral RT activity at 1, 3, 5 and 7 days post infection (dpi). The data shown represent the means and standard errors of the means from three separate experiments. *P<0.05, compared with empty vector transfected cells.

The ability of sELR1 to inhibit EIAV infection on target cells is the most important characteristic of the soluble receptor studied here. The recombinant sELR1 was overexpressed in 293T cells, and EIAV preparations pre-incubated with either the culture medium of sELR1-expressing cells or the culture medium of cells transfected with the empty pcDNA3.1 vector were used to infect eMDMs. The replication of the infected EIAV was then examined by measuring the viral RT activity. As shown in Fig. 4, EIAV incubated with the medium of pcDNA3.1-transfected cells and the untreated virus replicated at similar rates. In contrast, the virus that was pre-incubated with medium from sELR1-expressing cells showed a dramatically retarded replication that was characterized by delayed detectable RT activity and low rates of increase. This result strongly indicated that overexpressed recombinant sELR1 was able to inhibit EIAV infection in vitro, probably by competing with the membrane-associated receptor for the binding of virions on target cells. This result also suggests that the receptor encoded by ELR1-IN was a secreted protein.

### The inhibitory effect of sELR1 on EIAV infection of target cells

**Figure 5:**
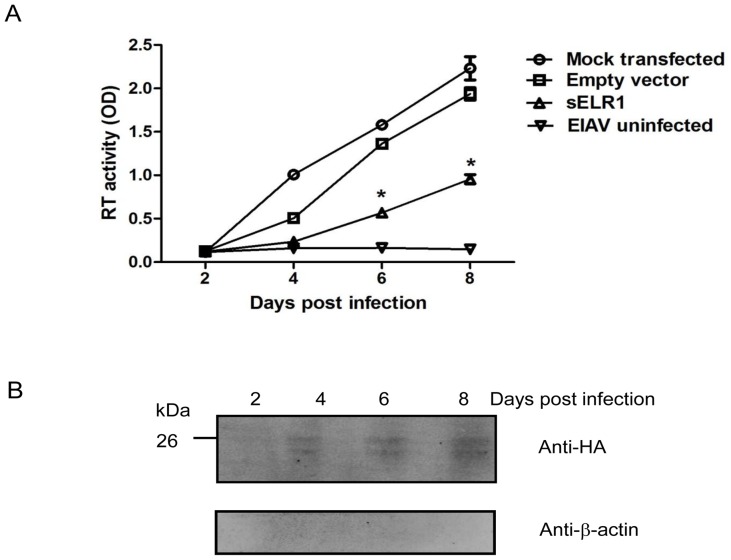
EIAV replication in FED cells overexpressing sELR1. The inhibitory effect of sELR1 on viral replication in infected cells was investigated. sELR1 was overexpressed in FED cells by transfection with the pcDNA3.1-ELR1-IN expression vector. These cells were infected with EIAV_DLV34_ 24 hours later. FED cells that were mock-transfected or transfected with the empty vector pcDNA3.1 were also infected with the virus as controls. (A) EIAV replication was monitored as viral RT activity at 2, 4, 6 and 8 dpi. The data shown represent the means and standard errors of the means from three separate experiments. *P<0.05, compared with empty vector transfected cells. (B) The presence of sELR1 in the culture medium of transfected FED cells were examined by Western blot at 2, 4, 6 and 8 dpi.

Because sELR1 interacted with EIAV in culture medium, significantly blocking the subsequent infection of target cells by the sELR1-preincubated virus, it was logical to examine whether the expression of the soluble receptor by the target cells interferes with viral infection. Thus, the soluble form of ELR1, tagged with HA, was overexpressed in FED cells, a major target of EIAV in vitro, by transfection with the sELR1 expression vector pcDNA3.1-ELR1-IN. The cells were then infected with EIAV 24 h after the transfection. The results revealed that EIAV replicated at a significantly slower rate in the FED cells transfected with the sELR1 expression vector than in the cells transfected with the empty vector at 6 and 8 dpi (*P*<0.05, see Fig. 5A), indicating the inhibitory effect of sELR1 expressed in the target cells upon EIAV infection. In addition, the presence of recombinant sELR1 in the culture medium was confirmed by Western blot using an anti-HA antibody. There was no β-actin detected in the medium, which ruled out the possibility that these soluble receptor molecules were leaked from broken cells (Fig. 5B).

### The effect of EIAV infection on ELR1-IN and ELR1 mRNA expression

**Figure 6:**
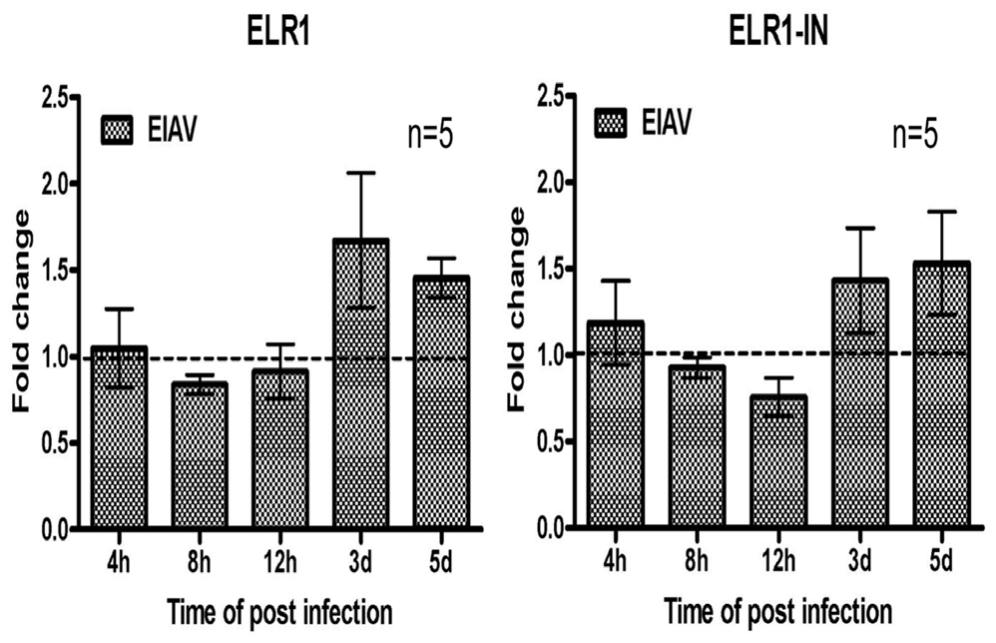
The regulation of membrane-associated and soluble ELR1 mRNA expression in eMDMs infected with EIAV. mRNA was extracted from eMDMs infected with DLV34 for 4, 8, 12(**A**) and ELR1-IN (**B**) were quantified by SYBR real-time RT-qPCR. The values were normalized to endogenous β-actin as a control. The fold increase was calculated based on the level of mRNA in the mock-infected cells. The data represent the means and standard errors of the means of five separate experiments.

As shown in Fig. 2, the level of ELR1 expression varied in different hosts and at different detection times. Thus, we tested the effect of EIAV infection on the expression of the membrane-associated and soluble forms of ELR1. The ELR1 and sELR1 mRNAs were quantified in eMDMs infected with EIAV for 4 hours to 5 days using SYBR Green real-time PCR with β-actin as the internal control. The fold changes of ELR1 and sELR1 mRNA (ELR1-IN) after EIAV infection at each time point are shown in Fig. 6, with the fold changes of the mRNA levels in mock-infected cells as the baseline (indicated as 1). Both the ELR1 and ELR1-IN levels showed no apparent change or a slight decrease during the 4 to 12 h post-infection. However, a notable increase (approximately 1.5-fold, *P*<0.05) was observed for the ELR1 and sELR1 mRNAs in cells infected with EIAV for 3 and 5 d when compared with the untreated controls.

## Discussion

Alternative splicing is an important posttranscriptional regulation mechanism in eukaryotic cells. Most genes in eukaryotes are alternatively spliced into several forms, and the percentage of these genes can be as high as 93% in humans [22]. Through such a mechanism, multiple proteins with different functions can be produced from a single gene, thus providing an opportunity for adaptation to different environments [23]. Multiple alternative splicing variants of ELR were identified in this study for the first time. The predominant isoform that produces a functional ELR1 is a species of 942 bp containing exons 1–9. The other two major isoforms include ELR1-IN, which contains an insertion of a fragment of intron 6, and ELR-DE, in which exon 3 is deleted following splicing. These three major forms of spliced mRNA exhibit distinct expression levels and ratios in different individuals and at different time points.

The inserted fragment in ELR1-IN was determined to be a fragment of an intron, and intron retention is considered to occur in unprocessed or incompletely spliced pre-mRNA [24], [25]. It is known that abnormal transcript variants, such as intron retentions, are occasionally generated in cells but normally do not exist long enough to have a functional impact. In addition, it has been reported that, although intron retention events are common in human genes, their frequency relative to the dominant transcript is generally low [22], [25]. Our results demonstrated that ELR1-IN differed from a typical intron retention transcript: it contained a fragment of an intron and appeared with a high frequency compared with the predominant transcript of ELR1 (approximately 25%). ELR1-IN was consistently detected in different individuals at different times, and it was translated into a functional soluble ELR1 when the recombinant molecule was expressed. In addition, we did not find the typical “GT/AG” and “GC/AG” splicing sites in these spliced ELR1 variants. However, due to the absence of a sufficient equine alternative splicing database, the splicing model of ELR1-IN remains unclear [24].

The protein encoded by ELR1-IN was confirmed to be a soluble receptor by several different approaches, including Western blot, live cell imaging, confocal microscopy and flow cytometry, which demonstrated that most of the receptor molecules were secreted out of the cell after being expressed. However, 3D live cell imaging and confocal microscopy showed that a small portion of sELR1 associated with the cell surface, although the fluorescent signal was much weaker than that of ELR1, the membrane-associated form of EIAV receptor. Because the predicted hydrophobic transmembrane domain is absent in the sELR1, the association of this soluble protein on cell membrane is mediated by the glycoside chains, like that observed for the EIAV gp90 surface protein [4], [5].

Because soluble viral receptors containing virus-binding motif(s) can be used as valuable tools for evaluating viral entrance into target cells, these molecules have played important roles in studies of virus-host interactions [26], [27]. It was reported that the co-incubation of soluble CD4 with HIV-1 envelope protein resulted in the dissociation of the gp120 surface protein and gp45 transmembrane protein [6], [28]. CD4 is also the cellular receptor of HIV-1, HIV-2 and simian immunodeficiency virus SIVmac and SIVagm. The soluble form of CD4 (sCD4) can block HIV-1 and HIV-2 infection in human lymphoma cell lines, but it enhances SIVagm infection in these cell lines by 10 to 100-fold [29]. A similar augmenting effect of soluble viral receptors on the invasion of viruses was also observed in studies on herpes simplex virus (HSV) [30], [31]. In addition, a study on Jaagsiekte retrovirus (JSRV) found that although the soluble form of its receptor, Hyal2 (sHyal2), did not mediate the entrance of a JSRV-pseudotype retroviral vector into cells lacking the integrated Hyal2, this purified soluble protein significantly inhibited the infection of the pseudotyped vector in the target cells of JSRV [13]. Moreover, some soluble receptors in the plasma can be considered markers of disease progression, such as soluble TNF receptor (sTNFR) II and soluble IL-2 and IL-6 receptors in HIV-1-infected patients [10], [14], [32]. Therefore, soluble receptors play important roles in the interactions between viruses and hosts [33]–[37]. Studies on the mechanisms regulating the formation of soluble viral receptors should lead to a better understanding of the cellular responses to invading viruses.

In this study, the soluble form of the EIAV receptor sELR1 markedly inhibited EIAV infection of its target cells when examined by in vitro competition studies, and this inhibitory effect appeared to increase with increasing infection time. Additionally, the expression levels of ELR1 and its alternative splicing variants were regulated by EIAV infection. In a separate study on the restriction of EIAV superinfection, we found that the up-regulation of sELR1 induced by the initially infected viral strain was correlated with protection against subsequent viral infection (unpublished data). At present, it remains unclear whether the inhibitory effect of sELR1 on EIAV infection is based on the competition of the binding of the virus to membrane-associated ELR1 or on the polymerization with EIR1, which may trigger or may reduce signal transduction of membrane-associated receptors, therefore interfering with the expression of downstream cellular proteins [6]. However, our data indicate that sELR1 is not merely a truncated product of a spliced ELR1 transcript but is a functional protein with important biological roles. ELR1 is a member of the TNFR superfamily, which is known to have important roles in regulating immune responses and inducing pathological outcomes [4], [38]. Previous studies have shown that plasma TNFα concentration is positively correlated with the clinical symptoms of EIAV-infected horses [18]. It was also reported that sTNFR was significantly elevated in patients with HIV-1 infection [10]. Because ELR1 acts as both the receptor of EIAV and a member of TNFR family, the finding that sELR1 is regulatable and can compete its membrane-associated prototype form implicates the involvement of this soluble protein in both of the viral infectivity and the host response, especially that TNFα is closely related with EIA symptoms. The possible role of sELR1 in ELR1 signaling is an interesting question to be explored.

The results of this study indicate that the ability to inhibit EIAV infection of target cells in vitro may be related to reduced viral entry into host cells in vivo. The regulated expression levels of both the soluble and membrane-associated forms of ELR1 by EIAV reveal the possible role of sELR1 in the interaction between virus and host. Therefore, sELR1 may be considered a secreted cellular factor that regulates susceptibility to EIAV and the pathological and immunological responses of the host. Furthermore, the identification of ELR1-IN and other alternative splicing variants of ELR1 suggests additional approaches for further studies on the pathogenesis and immunogenicity of EIAV infection.

## Supporting Information

Figure S1 **Recombinant sELR1 was expressed in 293T cells as a fusion protein with a GFP tag linked at the N-terminus.** The percentage of GFP-expressing cells and the fluorescence intensity of sELR1-GFP in cells were examined by flow cytometry. The GFP-tagged ELR1 was also expressed and analyzed in parallel in the experiments shown in this figure. All the experiments were performed for three times, and a representative result is shown.
(TIF)

Movie S1 **A 3D live cell imaging of the dynamic expression and distribution of the GFP-ELR1 and GFP-sELR1 was taken to examine the dynamic cellular distribution of sELR1 and compare with that of ELR1.** The images of sELR1 were enhanced at a higher level than ELR1.
(ZIP)
