# Supplementary figures and images for: The Soluble Form of the EIAV Receptor Encoded by an Alternative Splicing Variant Inhibits EIAV Infection of Target Cells
Source: PLoS One. 2013 Nov 22;8(11):e79299. doi: 10.1371/journal.pone.0079299 (PMC3838338; doi:10.1371/journal.pone.0079299)

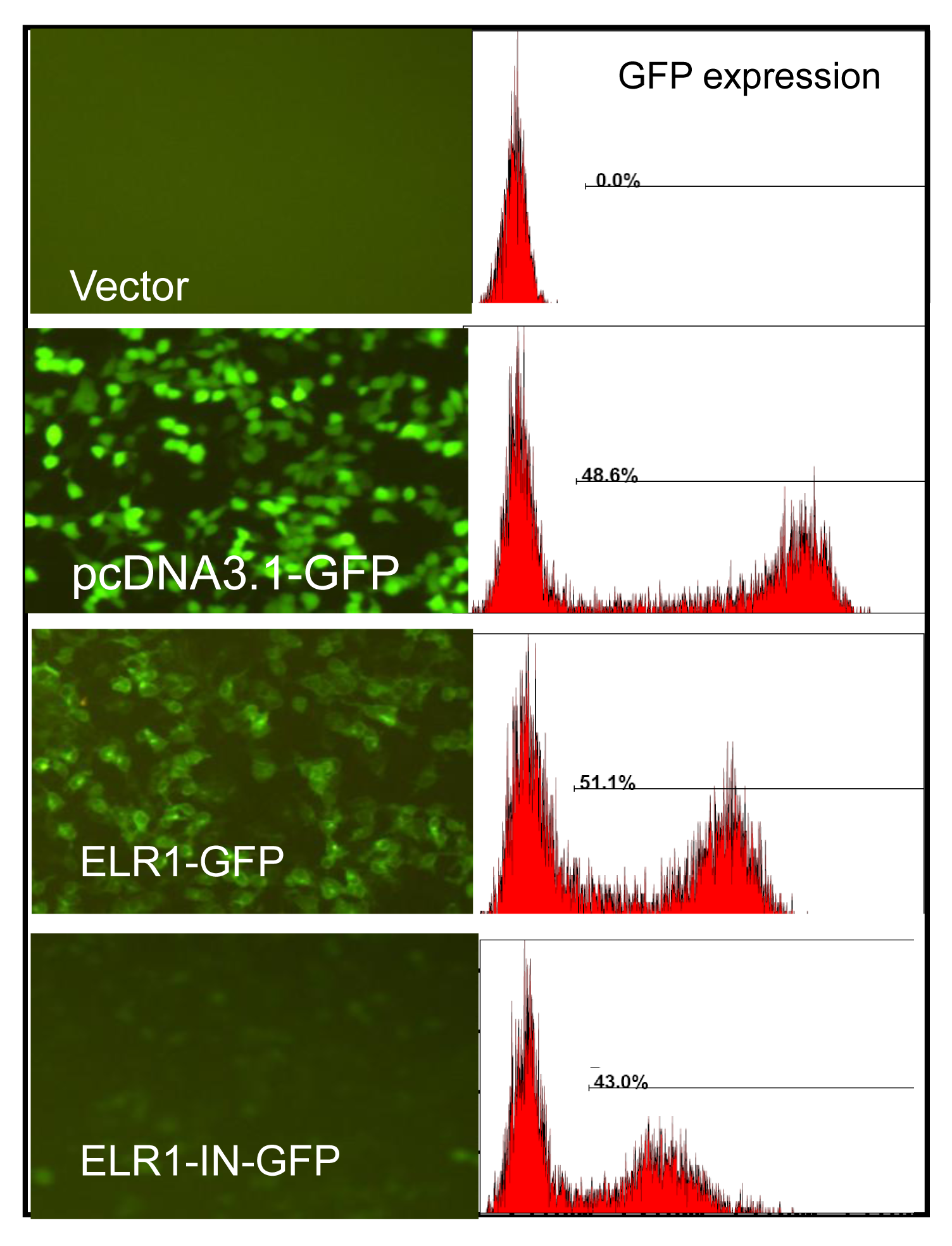

Supplement: Figure S1 — Recombinant sELR1 was expressed in 293T cells as a fusion protein with a GFP tag linked at the N-terminus. The percentage of GFP-expressing cells and the fluorescence intensity of sELR1-GFP in cells were examined by flow cytometry. The GFP-tagged ELR1 was also expressed and analyzed in parallel in the experiments shown in this figure. All the experiments were performed for three times, and a representative result is shown. (TIF) [file pone.0079299.s001.tif]
